# Nitric Oxide Signalling Augments Neuronal Voltage-Gated L-Type (Ca_V_1) and P/Q-Type (Ca_V_2.1) Channels in the Mouse Medial Nucleus of the Trapezoid Body

**DOI:** 10.1371/journal.pone.0032256

**Published:** 2012-02-28

**Authors:** Adam J. B. Tozer, Ian D. Forsythe, Joern R. Steinert

**Affiliations:** Neurotoxicity at the Synaptic Interface, MRC Toxicology Unit, University of Leicester, Leicester, United Kingdom; Dalhousie University, Canada

## Abstract

Nitric Oxide (NO) is a diffusible second messenger that modulates ion channels, intrinsic excitability and mediates synaptic plasticity. In light of its activity-dependent generation in the principal neurons of the medial nucleus of the trapezoid body (MNTB), we have investigated its potential modulatory effects on native voltage-gated calcium channels (Ca_V_) within this nucleus. Whole-cell patch recordings were made from brain slices from P13–15 CBA mice. Slices were incubated with the inhibitor of neuronal nitric oxide synthase (nNOS) 7-nitroindazole (10 µM) and pharmacological blockers used to isolate Ca^2+^ current subtypes. Unpaired observations in the presence and absence of the NO-donors sodium nitroprusside (SNP, 100 µM) or Diethyl-ammonium-nonoate (DEA, 100 µM) were made to elucidate NO-dependent modulation of the expressed Ca_V_ subtypes. A differential effect of NO on the calcium channel subtypes was observed: Ca_V_1 and Ca_V_2.1 (L+R- and P/Q+R-type) conductances were potentiated, whereas N+R-type (Ca_V_2.2) and R-type (Ca_V_2.3) current amplitudes were unaffected. L+R-type currents increased from 0.36±0.04 nA to 0.64±0.11 nA and P/Q+R-type from 0.55±0.09 nA to 0.94±0.05 nA, thereby changing the balance and relative contribution of each subtype to the whole cell calcium current. In addition, N+R-type half-activation voltage was left shifted following NO exposure. NO-dependent modulation of P/Q+R and N+R-type, but not L+R-type, channels was removed by inhibition of soluble guanylyl cyclase (sGC) activity. This data demonstrates a differential effect of NO signalling on voltage-gated calcium entry, by distinct NO-dependent pathways.

## Introduction

Nitric oxide is an important signalling molecule and volume transmitter with a functional role in both central and peripheral systems [1], [2], [3], [4]. The modulation of ion channels is one of several mechanisms mediating the physiological functions of NO, including relaxation of vascular smooth muscle [5], [6], neurotransmission, synaptic plasticity and neurodegenerative processes [1], [4], [7], [8]. Ion channel modulation by NO can be mediated directly by S-nitrosylation [9], [10] or indirectly through second messengers involving the activation of soluble guanylyl cyclase (sGC) to generate cyclic guanosine monophosphate (cGMP) [8], [11]. The presence of nNOS and sGC in the auditory brainstem (including the MNTB) and its functional role in modulating potassium channels and excitability within the auditory brainstem has been documented [4], [12].

Activity-dependent Ca^2+^-signalling is vital throughout the brain with voltage-gated Ca^2+^ entry being mediated by multimeric Ca^2+^ channels [13], [14]. Ca_V_ channels are grouped into three families according to the genetic relatedness of the pore-forming α1 subunit, which confers the voltage sensitivity and conductance specificity of the channels [15]. In mouse MNTB four high voltage activated subtypes are expressed in maturing (P>12) principal neurons, L-type (Ca_V_1), P/Q-type (Ca_V_2.1), N-type (Ca_V_2.2) and R-type (Ca_V_2.3) [16] which do not contribute equally to the whole-cell current.

Here we show that NO-dependent potentiation of Ca_V_1 and Ca_V_2.1 channel amplitudes and a hyperpolarising shift of half-activation voltages of Ca_V_2.2 by distinct pathways underlies the increase in whole-cell current amplitude.

## Results

### Nitric oxide augments whole-cell barium currents

**Figure 1:**
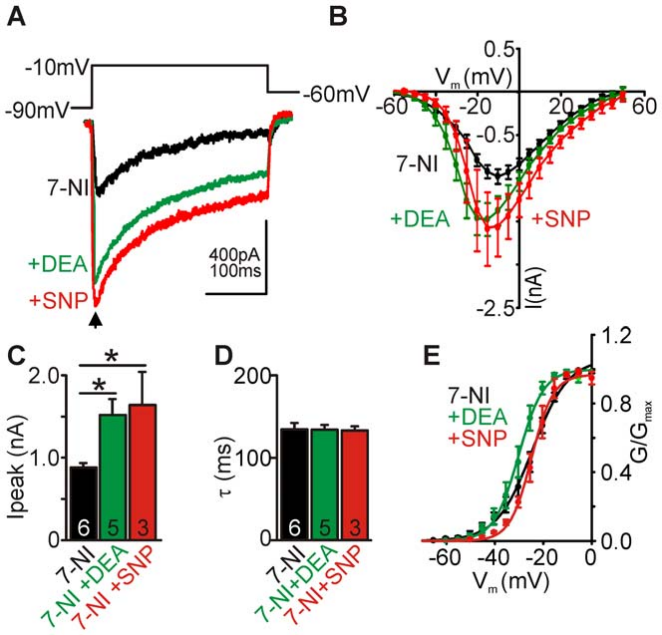
Nitric oxide augments whole-cell barium currents. **A**, Raw traces of recorded unpaired barium currents of control (7-NI) neurons and following exposure to NO donor (DEA or SNP, 100 µM each) using the protocol shown above. **B**, Average I/V curves under conditions indicated. **C**, Mean peak Ca_V_ currents (arrow in A) show NO-dependent augmentation induced by sodium nitroprusside (SNP, 100 µM) or Diethyl ammonium-nonoate (DEA-nonoate, 100 µM), data denote mean±SEM, unpaired observations, Student's *t*-test, *P<0.05. **D**, Mean inactivation kinetics (τ) of currents evoked by step depolarisation to −10 mV. **E**, Boltzmann fit to conductances (G/G_max_) under conditions indicated.

In physiological aCSF containing 2 mM Ca^2+^ we found total I_Ca_ amplitudes in the range of 400 pA (not shown) consistent with previous reports [16]. The use of Ba^2+^ improved current resolution as peak amplitudes of whole-cell currents increased over 2-fold in the presence of Ba^2+^ compared to Ca^2+^, thus the following experiments were performed using Ba^2+^ as the charge carrier. In order to assess nitrergic effects on Ca_V_ we applied NO-donors to slices where residual modulation of Ca_V_ by NO was removed by pre-incubation with the nNOS inhibitor 7-Nitroindazole (7-NI, 10 µM). Raw traces from one control cell (black trace), and two from cells treated with the NO donors: Diethyl ammonium-nonoate (DEA, 100 µM, green trace) and sodium nitroprusside (SNP, 100 µM, red trace), respectively are shown in Figure 1A. Average (mean±sem) current voltage (IV) relationships for neurons under control (7-NI-treated) and NO-donor treated conditions are shown in Figure 1B. NO led to similar whole–cell current potentiation induced by either donor: 7-NI control peak amplitude was 0.98±0.09 nA (n = 6); DEA was 1.5±0.2 nA (n = 5, P<0.05) and SNP was 1.6±0.4 nA (n = 3, P<0.05) (Figure 1C). We did not observe any changes in inactivation rates (τ) of the total current following NO treatment: 7-NI τ was 134±8 ms; DEA τ was 134±6 ms; SNP τ was 133±5 ms (Figure 1D). Half-activation voltages (V_1/2_) which were calculated by fitting a Boltzmann function to the normalised conductance (G/G_max_) (Figure 1E) did not show any differences between control and NO-donor treatment: 7-NI V_1/2_ was −25.5±1.5 mV (n = 6); DEA V_1/2_ was −30.4±2.0 mV (n = 5); SNP V_1/2_ was −23.5±1.5 mV (n = 3)). Overall, the data showed that treatment of 7-NI incubated slices with the NO-donors significantly potentiated whole-cell I_Ba_ without affecting channel inactivation kinetics or half-activation voltages.

### NO differentially modulates natively expressed Ca_V_, potentiating L- (Ca_V_1) and P/Q-type (Ca_V_2.1) currents

**Figure 2:**
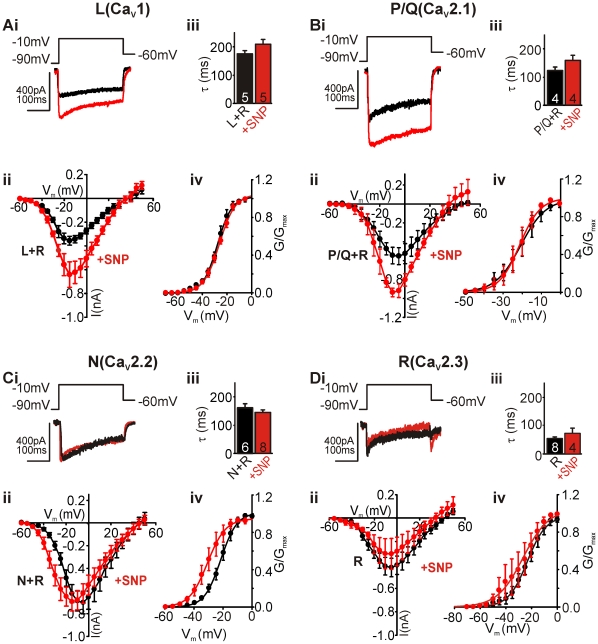
NO differentially modulates expressed Ca_V_s, potentiating L (Ca_V_1) and P/Q-type (Ca_V_2.1). **A-Di**, Raw traces of pharmacologically isolated currents (+R-type) in **A-Ci** (black trace), and the same current from cells treated with SNP (red). Traces generated by depolarising steps from −90 mV to −10 mV (holding potential of −60 mV). **Aii–Dii**, Average IVs of control (black circles) and SNP-treated (red circles) neurons. **Aiii–Diii**, Summary bar graph showing mean inactivation kinetics (τ) of currents in control (black) and SNP-treated (red) conditions. **Aiv–Div**, Mean activation curves of currents recorded from untreated (black) and SNP-treated (red) cells. Curves are fitted to conductances applying a Boltzmann equation.

We next investigated how the four Ca_V_ subtypes underlying the whole-cell current were affected by NO. Using pharmacological tools we isolated the four Ca_V_ subtypes contributing to the whole-cell current (Figure 2) in mouse principal MNTB neurons: L-, P/Q-, N- and R-type. R-type current remained in all scenarios as no specific blockers of this channel were available [17].

L+R-type and P/Q+R-type channels were isolated pharmacologically by blocking N- and P/Q-type channels or L- and N-type channels, respectively (see Methods for details). Examples of raw control traces (black), and in the presence of NO-donor SNP (100 µM, red) generated by a step depolarisation from −90 mV to −10 mV are shown in Figures 2Ai and Bi. Current voltage (IV) relationships of the averaged currents (mean±sem), generated from a voltage step protocol that included a hyperpolarising pre-pulse to −90 mV before 5 mV step depolarisations (300 ms) to 50 mV from a holding potential of −60 mV, are shown in Figures 2Aii and Bii. From both IVs it is clear that NO-treated cells (red circles) produce larger currents on depolarisation than control cells (black circles). NO exposure potentiated L+R-type and P/Q+R-type current amplitudes without affecting channel inactivation kinetics: L+R-type τ was 179±10 ms and SNP-treated τ was 214±18 ms; P/Q+R-type τ was 123±12 ms and SNP-treated τ was 158±15 ms (Figure 2Aiii and Biii). NO exposure also had no effect on the voltage dependence of current activation: L+R-type half-activation voltage (V_1/2_) was −26.5±1.7 mV and SNP-treated V_1/2_ was −26.1±1.3 mV; P/Q+R-type V_1/2_ was −19.7±4.1 mV and SNP-treated V_1/2_ was −21.5±3.0 mV (Figure 2Aiv and Biv).

In contrast, when isolating N+R- and R-type channels we did not detect any effects of NO on either amplitude or channel inactivation kinetics. Raw traces from control and NO-treated cells generated by a step depolarisation to −10 mV showed no effect of NO on the isolated N+R-type and R-type currents (Figure 2Ci and Di). The averaged IVs (mean±sem) of the isolated N+R- and R-type channels generated by 5 mV step depolarisations (as described above) again showed no effect of NO on the current amplitudes (Figure 2Cii and Dii), but did reveal a leftward shift in N+R-type activation voltage. Channel inactivation kinetics for either subtype were not affected by NO: N+R-type control τ was 158±18 ms and SNP-treated τ was 146±9 ms; R-type control τ was 55±6 ms and SNP-treated τ was 74±19 ms (Figure 2Ciii and Diii). The N+R-type activation curve confirmed the leftward shift in the voltage dependence of activation of the channels: control V_1/2_ was −20.9±2.8 mV, whereas SNP-treated V_1/2_ was −29.7±2.6 mV (P<0.05, Figure 2Civ). However, no significant shift in V_1/2_ was observed for the isolated R-type channels: control V_1/2_ was −21.7±3.1 mV and SNP-treated V_1/2_ was −28.2±6.0 mV. This suggests that only the voltage dependence of activation of the N-type channels is leftward shifted by NO.

Taken together these data demonstrate a NO-induced potentiation of L- and P/Q-type currents, without affecting their channel inactivation kinetics or half-activation voltages, and a leftward shift in the half-activation of N-type channels, but no effect on N-type current amplitude.

### NO-dependent modulation of channel subtypes is maintained over time

**Figure 3:**
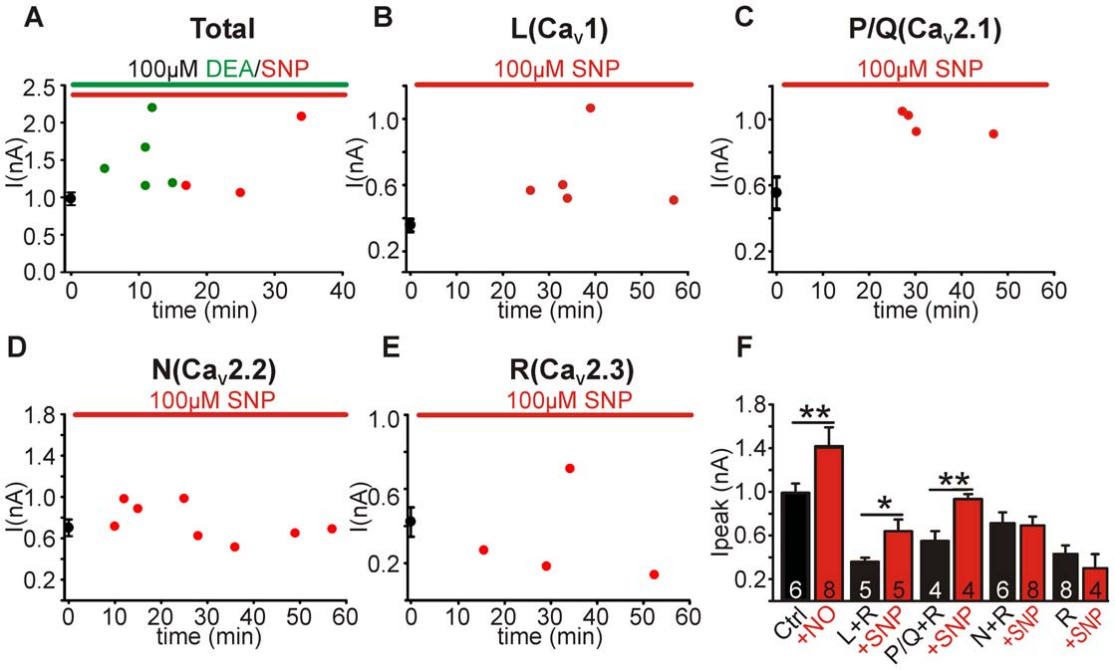
NO-dependent modulation of channel subtypes is maintained over time. **A**, Peak whole-cell I_Ba_ amplitudes from unpaired recordings, control (black , mean±SEM), SNP (red)- and DEA (green)-treated neurons. **B**, Peak L-type (+R-type) current amplitudes from unpaired recordings made from control (black, mean±SEM) and SNP-treated (red) neurons. **C**, Peak P/Q-type (+R-type) current amplitudes from unpaired recordings made from control (black, mean±SEM) and SNP-treated (red) neurons. **D**, Peak N-type (+R-type) current amplitudes from unpaired recordings made from control (black, mean±SEM) and SNP-treated (red) neurons. **E**, Peak R-type current amplitudes from unpaired recordings made from control (black, mean±SEM) and SNP-treated (red) neurons. **F**, Contribution of the different Ca_V_ subtypes to whole-cell currents under control (black) and nitrergic conditions (red). R-type current was present throughout all recordings. Note the large proportional increase in L- and P/Q-type currents following NO exposure. Data denote mean±SEM, n = number of neurons, Student's *t*-test, *P<0.05, **P<0.01.

As we have previously shown that nitrergic effects can vary in their time course [4], [18] we next sought to analyse the time dependence of NO effects on Ca^2+^ channels. The averaged control (black circles, mean±sem) and whole-cell peak current amplitudes from unpaired recordings from slices exposed to NO over time (red circles) are shown in Figure 3A–E. We found that the potentiation of total whole-cell, L+R-type and P/Q+R-type currents observed in the previous figures was maintained over 5–60 min of NO exposure (Figure 3A–C). Similarly, the null-effect of NO on N+R- and R-type current amplitudes was also maintained over the 60 min of NO exposure (Figure 3D, E). Due to the lack of a temporal aspect to the NO-dependent modulation of the currents we summarised the observed current amplitude changes in the bar graph in Figure 3F. The bar graph shows the potentiating effect of the pooled NO donors (100 µM DEA and 100 µM SNP) on the whole-cell I_Ba_: control 7-NI treated peak I_Ba_ was 0.98±0.09 nA (n = 6) and NO treated was 1.52±0.18 nA (n = 8, t-test, P<0.001). It also shows the potentiation of L+R- and P/Q+R-type currents by the NO donor SNP (100 µM): L+R-type control was 0.36±0.04 nA (n = 5) and was increased to 0.64±0.11 nA (n = 5, P<0.05); P/Q+R-type control was 0.55±0.10 nA (n = 4) and was increased to 0.94±0.05 nA (n = 4, P<0.01). N+R- and R-type current amplitudes were unaffected in cells treated with SNP (100 µM): N+R-type control was 0.70±0.10 nA (n = 6) and N+R-type +NO was 0.69±0.08 nA (n = 8); R-type control was 0.42±0.08 nA (n = 8) and R-type +NO was 0.30±0.13 nA (n = 4, Figure 3F).

Previous data from mouse MNTB neurons [16] and also rat cerebellar granule neurons [19] showed similar relative subtype current contributions to whole-cell Ca^2+^ currents as we reported here. The lack of modulation of N- and R-type current amplitudes means that L- and P/Q-type currents underlie the NO-dependent potentiation of the whole-cell I_Ba_. NO signalling dramatically increased L- and P/Q-type channel contributions to whole-cell I_Ba_ relative to control where N- and R-type seem to be the dominant currents (Figure 3F).

### Soluble guanylyl cyclase activity is necessary for P/Q-type, but not L-type potentiation

We next sought to delineate the pathway by which L- and P/Q-type channel amplitudes and N-type activation voltage dependence are modulated. Cells were treated with the NO-donor in the presence of the sGC inhibitor ODQ (1 µM) and the modulated Ca_v_ channel subtypes were isolated pharmacologically as above.

**Figure 4:**
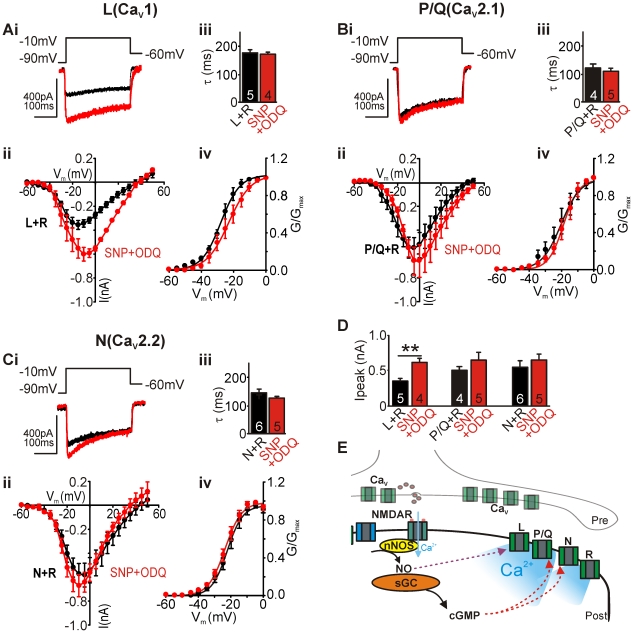
Soluble Guanylyl Cyclase (sGC) activity is necessary for L- and P/Q-type potentiation. **Ai–Ci**, Raw traces of control L- and P/Q-type currents (+R-type, black), and the same currents from SNP treated cells in the presence of ODQ (red). Traces generated by depolarising steps from −90 mV to −10 mV (holding potential of −60 mV). **Aii–Cii**, Average IVs of control (black circles) and ODQ+SNP-treated (red circles) neurons. **Aiii–Ciii**, Bar graph shows mean inactivation kinetics (τ) of currents in control (black bar) and ODQ+SNP-treated (red) neurons. **Aiv–Civ**, Mean activation curves of control (black) and ODQ+SNP-treated (red) neurons. Curves are fitted using a Boltzmann equation. R-type current was present throughout all recordings. **D**, Summary bar graph of mean peak amplitudes of control (black) and ODQ+SNP-treated (red) conditions. Data denote mean±SEM, n = number of neurons, Student's *t*-test, **P<0.01. **E**, Schematic illustration showing differential NO signalling to L- (direct NO effects), P/Q- and N- type (sGC-mediated effects) Ca^2+^ channels, resulting in augmented Ca^2+^ influx following nitrergic activation.

Control traces of P/Q+R-type and L+R-type currents are shown in Figure 4Ai, Bi (black), respectively, and in the presence of NO-donor SNP (100 µM) together with the sGC blocker ODQ (1 µM, red). NO-dependent potentiation of P/Q-type but not L-type channel amplitudes was removed by ODQ (Figure 4Aii, Bii, SNP+ODQ: L+R-type: 0.62±0.06 nA (n = 5, P<0.01); P/Q+R- type: 0.70±0.12 nA (n = 4)); N-type current amplitudes in the presence of SNP were not affected by ODQ (0.67±0.09 nA (n = 8)). This data illustrates a differential effect of nitrergic modulation on the recorded Ca_V_ channel subtypes. Additionally, the NO-induced left-shift in N-type activation was removed in the presence of ODQ (Figure 4Civ, V_1/2_: −22.2±1.3 mV), whereas half-activation voltages of P/Q+R- and L+R-type currents were not affected by ODQ in the presence of SNP (Figure 4Aiv, Biv; P/Q+R: −21.1±3.0 mV; L+R: −22.2±2.9 mV). The bar graph in Figure 4D summarises the effects of ODQ on NO-induced changes on Ca_V_ channel amplitudes. ODQ had no effect on Ca_V_ channel inactivation kinetics of all subtypes (Figure 4Aiii–Ciii, SNP+ODQ: L+R-type τ, 171±7 ms; P/Q+R-type τ, 109±10 ms; N+R-type τ, 128±6 ms).

## Discussion

Here we have shown that activation of the NO signalling in mouse MNTB neurons enhanced L- and P/Q-type current but not N- and R-type current amplitudes and this subsequently causes a change in relative contribution of the four expressed subtypes to the total whole-cell current following nitrergic signalling. Intracellular Ca^2+^ is critically involved in many aspects of the neuronal “life cycle”, including communication, plasticity, development, differentiation, migration and cell death. Moreover, alterations in intracellular Ca^2+^ signalling pathways have been implicated in neurological diseases, such as migraine, epilepsy, ischemia, cerebral hemorrhage and Alzheimer's disease [20]. Together with abnormal nitrergic signalling reported in neurodegenerative conditions [7] these findings could be inter-connected. NO has a ubiquitous role in health and disease of the central nervous system [7] and, perhaps not surprisingly, NO is fundamentally involved in many physiological and pathological aspects of Ca^2+^ signalling in the neuron-glial network [8], [21], [22].

Ca^2+^entry into principal MNTB neurons occurs primarily via activation of postsynaptic AMPAR and NMDAR [4], [23], [24] but secondarily via depolarisation-induced activation of Ca_V_. Previously we have shown that NO can modulate postsynaptic AMPAR and NMDAR in addition to voltage-gated K^+^ channels (K_V_3.1, K_V_2.2) [4], [18], [23]. This broad nitrergic action ultimately affects postsynaptic neuronal excitability and information transmission. Whether similar NO signalling pathways occur presynaptically to modulate K_V_ and Ca_V_ channels or affect transmitter recycling/release remains to be elucidated. Importantly, all our recordings were performed at 37°C in unpaired experimental conditions to minimise cell dialysis thereby providing intact signalling required for the nitrergic pathways. Our results therefore represent a physiological approach where native Ca_V_ were differentially modulated by intact NO signalling.

Previous studies in native neurons, dissociated hair cells from frog and rat [25], [26], found that nitrergic activation reduced L-type and whole-cell I_Ca_ in a cGMP-dependent manner suggesting that NO regulation differs greatly between cellular systems. Different studies in rat cultured hippocampal [27] or cortical neurons [28] suggested a NO-mediated increase in L-type currents. N-type currents are suppressed following activation of the classical NO/cGMP/PKG pathway in neuroblastoma IMR32 cells due to a reduction in single channel open probability [29]. In contrast, P/Q-type channels, which are found throughout the brain and are postulated to participate in transmitter release, are augmented by NO in BHK cells [30]. Many of the differences reported above can be accounted for by differing signalling cascades: some NO effects are mediated via cGMP; others act via NO's ability to generate free radicals or produce peroxynitrite leading to protein S-nitrosylation or nitrotyrosination [7]. Redox-modulation of L-type channels has been reported in cardiac myocytes leading to current inhibition but this effect was NO-independent and mediated by CO [31].

As illustrated in Figure 4E, our data suggest that long-term exposure to NO, as seen during periods of enhanced neuronal activity leads to augmented Ca^2+^ influx via L- and P/Q-type Ca^2+^ channels which could have important downstream signalling effects as various Ca^2+^ channel subtypes are linked to multiple signalling pathways leading to changes in gene expression through: CREB (Ca^2+^ response element binding protein), CaRF (the Ca^2+^ response factor) and NFAT (the nuclear factor of activated T-cells) [32], [33]. Ca^2+^-induced gene expression has been linked to changes in intrinsic excitability mediated by L-type Ca^2+^ channels as reported in primary hippocampal cultures [34] or organotypic cultures of the auditory brainstem [35], whereas N-type Ca^2+^ channel activation leads to gene induction through PKA and PKC pathways. It is important to understand how nitrergic activity might influence these Ca^2+^-dependent pathways. NO-mediated augmentation of P/Q- and L-type channels adds to the versatile network of nitrergic signalling between cellular systems.

## Materials and Methods

### Brain slices preparation

Slices were prepared from CBA/Ca mice (P13–15), which were killed by decapitation in accordance with local animal's and the UK Animals (Scientific Procedures) Act 1986. All procedures were carried out complying with the policies and regulations according to the guidelines laid down by the MRC's animal's ethics and welfare committee (University of Leicester, UK) approved under the Home Office Project License 80/2100. Brainstem slices containing the Superior Olivary Complex (SOC) were cut in the transverse plane using a 7550 MM Integraslice (Campden, UK) at 200 µm at ∼0°C. Brain slices were incubated at 37°C in aCSF for an hour, before being kept at room temperature prior to experiments as described previously [36] in the presence of 10 µM 7-nitroindazole (7-NI).

### Electrophysiology

Cells were visualised by an upright Eclipse E600FN Microscope (Nikon, Japan) using 60× objective. Whole-cell voltage clamp patch recordings were obtained from principal neurons of the MNTB at 37°C as described previously [36]. Patches with a series resistance >20 MΩ or leak current >200 pA were excluded. Voltage clamp protocols (holding potential of −60 mV and steps of 5 mV increments for up to 500 ms) were generated using pClamp 10.2 software and applied by an Axopatch 200B amplifier with a Digidata 1322A interface (Axon Instruments, Molecular Devices, US). Data were sampled at 50 kHz and filtered at 10 kHz. Temperature was maintained using a CI7800 temperature perfusion controller and bath (Campden, UK).

### Ca_V_ experiments

Current-voltage relationships (IVs) were generated by plotting peak current against command potential. Final whole-cell access resistance was <20 MΩ and series resistance was compensated by 70% (10 µs lag). Pipette solution contained (mM): CsCl (120); NaCl (10); TEA-Cl (10); EGTA (1); HEPES (40); Phosphocreatine (5); Mg-ATP (2); Na-GTP (0.3); ZD7288 (0.01). External aCSF solution contained (mM): NaCl (95); NaHCO_3_ (26.2); TEA-Cl (30); BaCl_2_ (5); MgCl_2_ (1.3); KCl (2.5); glucose (10); NaH_2_PO_4_ (1.25); ascorbic acid (0.5); 7-NI (0.01). TTx (0.5 µM) was used externally to block sodium channels. All chemicals were obtained from Sigma, except for Ca^2+^ channel blockers. All recordings were leak subtracted offline by assessing the linear leak between −110 and −70 mV for generating I/Vs. Raw traces are un-manipulated other than removal of capacitance artefacts for better visualisation.

All external solutions contained blockers of synaptic inputs to prevent any spontaneous events (µM): DNQX (10), strychnine (1), bicuculline (10) (non-methyl derivate) and Ca^2+^ channel blockers as indicated (µM): Nifedipine (10), ω-Agatoxin IVA (0.2) (Ascent Scientific, UK), ω-Conotoxin GVIA (2) (Cambridge Biosciences, UK).

### Calcium channel isolation

L-type channels were isolated pharmacologically by blocking P/Q- and N-type channels with 2 µM ω-Conotoxin GVIA and 200 nM ω-Agatoxin IVA, respectively. P/Q-type channels were isolated pharmacologically by blocking L- and N-type channels with 10 µM Nifedipine and 2 µM ω-Conotoxin GVIA, respectively. N-type channels were isolated pharmacologically by blocking L- and P/Q-type channels with 10 µM Nifedipine and 200 nM ω-Agatoxin IVA, respectively. R-type channels were pharmacologically isolated by blocking L-, P/Q- and N-type channels with 10 µM Nifedipine, 200 nM ω-Agatoxin IVA and 2 µM ω-Conotoxin GVIA, respectively.

Table 1 shows the peak current data for all the Ca_V_ subtypes in control and NO-treated conditions. Our observed whole cell I_Ba_ obtained from our recordings = R+N+L+P/Q. However our estimated current size based on our recording scenarios is calculated as R+N+R+L+R+P/Q+R-3R and this = 0.767 nA. If we propagate the error (Δ) associated with each of the recording scenarios by using the formula:
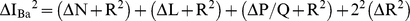


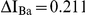
Our estimated I_Ba_, 0.77±0.21 nA falls in a similar range to our observed 0.98±0.09 nA. The table shows similar results for the NO-treated scenarios.

**Table 1:** Ca_V_ Subtype contribution to whole cell current.

	−NO		+NO	
Channel	Current (nA)	SEM	Current (nA)	SEM
**R**	0.422	0.079	0.303	0.129
**N+R**	0.702	0.100	0.694	0.081
**L+R**	0.356	0.037	0.641	0.107
**P/Q+R**	0.553	0.090	0.937	0.045
**Estimated I_Ba_**	0.767	0.211	1.666	0.293
**Observed I_Ba_**	0.980	0.085	1.519	0.175

### Statistics and Data Analysis

Statistical analyses utilized unpaired two-tailed Student's *t*-test. *P<0.05, **P<0.01 was considered significant. All data sets were tested for normality distributions prior to comparisons. Data denote mean±SEM, n - number of neurons tested. Activation parameters were determined by a Boltzmann function: I = I_max_/(1+exp(V−V_1/2_/k) with variables I_max_, V_1/2_ and *k* (slope factor). Inactivation data was obtained by fitting a single exponential decay to the current recorded at −10 mV.

All fits were performed using Clampfit 10.2, Sigmaplot (Systat) or Excel (Microsoft) with least squares minimization.
